# Prescribing in the Dark

**Published:** 1893-04-08

**Authors:** 


### The Hospital

An Institutional Journal of

Science, Medicine, Nursing, and Philanthropy.

*For Hospitals, Asylums, Infirmaries, Dispensaries, Medical Practitioners, Students, Nurses, and the Charitable Public.*

Vol. XIV.—No. 341.] April 8, 1893.

### Prescribing in the Dark.

During the past few years we have been in receipt of an increasing number of letters asking us to prescribe, to criticise prescriptions given by medical men, or to recommend particular physicians, surgeons, specialists, and the like. We do not for a moment suppose The Hospital is singular in having had experiences of this kind. No doubt every medical paper could tell a similar story, and no doubt other papers have felt it necessary from time to time to deal with the question in a decided and comprehensive way. It is hardly necessary for us to say that The Hospital never prescribes, that it never criticises prescriptions given by medical men to patients, and that it never recommends to enquirers, either physicians, surgeons, specialists, or general practitioners. A rule like this could be set forth in a single paragraph; and we should not think of wasting the space of a leader upon it. But the facts which have, with ever increasing persistence, been forced upon our notice, have generated the conviction that there is a good deal of "prescribing in the dark" carried on, both by the regular medical profession, and by its irregular camp followers. Now, in our judgment, prescribing in the dark is, and must be, dangerous; it is for the most part useless, and it is certainly immoral. For these reasons the practice demands exposure and ought to be promptly stamped out.

What do we mean by prescribing in the dark? One or two examples will show. A patient sends a friend to a medical man, perhaps to a family doctor, perhaps to a specialist, perhaps to a popular physician or surgeon. The friend's duty is to detail the patient's symptoms as he understands them, and to answer any questions the medical man may think it proper to ask. Perhaps the friend takes with him a sample of the patient's urine. That is one method of seeking a prescription in the dark. Another, and more frequent method, is for the patient himself, or more commonly herself, to write to a selected physician or specialist, and to give a "full, true, and particular account" of every symptom of ill-health which she can remember, imagine, or invent. The great thing to do is to "tell the doctor everything." All this sounds natural and entirely right; and if the methods employed could by any possibility lead to the desired results of the patient's advantage and restoration to health, we should have nothing to say against them. But we affirm with confident assurance that, except by accident and good luck, it is not possible for any physician or specialist to render the least degree of useful service to any patient by the pursuit of such methods as are here described.

But why may not a patient in the country write to a doctor in London, describe her symptoms, and say exactly what she requires just as easily as she can write to a draper for cloth of a particular kind and colour, or to her dressmaker for a gown of a certain fashion and form? The answer is, "Because she cannot." The very nature of the case renders the problem insoluble by any such means. Here is the question in a nutshell. The patient insists above all things that the doctor must know what she feels: the doctor insists, or ought to insist, that he shall not only know what the patient feels, but also exactly how she is. He must know how she is anatomically, physiologically, and pathologically; and on these points the patient is no more capable of enlightening the doctor by letter or special messenger than she is of telling the colour of his beard, which she has never seen.

"But why make a fuss and a pother about such a subject?" says a *laissez faire* critic. "Can the doctor not satisfy a foolish woman's desire for change by ordering her some harmless combination, pocketing his couple of guineas, and saying nothing about it?" He can, and probably he often does. But is work of this kind honest? Let us pull ourselves together here! Is medicine a calling of honesty, of science, of hard fact? Now, either it is or it is not! If it be, then the cynical approval, or the cynical avoidance of disapproval, of methods of this kind savours strongly of the acting of a lie, and a very base one. In everything we must have a standard of honesty, and the standard may be either absolute or relative. We may decide to be honest in the highest degree, or we may decide to be "as honest as we can." Science is honest; there is no denying that fact. The man who has had a medical education has acquired the habit of seeing things exactly as they are, and of describing them exactly as they are. When, therefore, a medical man adopts for himself the standard of relative honesty, of being "as honest as he can" instead of that of absolute honesty, he himself knows, whoever does not, that he is at heart a knave, and not worthy of the confidence of truthful and candid men and women.

We have put the case strongly, and that not merely in the interests of morality, but in the interests of medicine as a science and an art. The man who claims for himself the right to practise medicine with merely relative honesty will not be long before he claims the parallel right of practising it with merely relative skill. He will be content with that which is second best. As his practice increases, and it will increase if he be a man of tact and pleasant manners, he may sink down to contentment with the third or fourth best. When he has reached this stage his patients have little to hope for from him, and his art and profession nothing at all. His morals and his art have undergone putrefactive changes; he is an injury and a deadly poison in the corporate medical organism.

This is the kind of man who "prescribes in the dark." His rule is to prescribe as often as he has the opportunity; that is, if the guineas are duly forthcoming. Such a rule may appear to many to be quite sound. The medical man's art is his business; and why should a man not do business when opportunity offers? It is one of the maxims of business to be always ready to traffic and never to refuse "hard cash." But every art must be consistent with itself; every calling in life must be governed by rules proper to itself. The man who paints pictures paints, for his art's sake, the very best he can: he would be no better than a lunatic if he did not. Similarly the poet rises to his highest efforts on all possible occasions. The essential thing about medicine, as about painting and poetry, is not money-making; it is patient-curing. In this it differs entirely from commerce. The end and aim of commerce is to make money; the making of money is an accident in medicine. The real aim and business of the physician is the health and happiness of his patient; that and nothing else. But must not the physician live? True! And when he has practised his art with the highest degree of skill, he will insist upon his just dues in the way of payment. But, payment or no payment, his art is his art, and that must on every occasion be practised with all the best powers, all the honesty, and all the resources of knowledge and experience which he has at command.

Such is the normal rule of life for the medical man; and such a rule and standard no man can work up to who consents to practice in the dark. It may be said that the standard is impossibly high. Whoever takes that ground shows at once that his scientific studies have found in him no aptitude for science. What mistress can compare with science for sternness and absoluteness? Not morality, not religion makes higher demands than science, than medicine which is a part of science. No man can be at one and the same time a scientific physician and a second-rate moralist. The truest truth, the most real reality, the thing as it is, and nothing else, is what scientific medicine demands of every man. To practice after this fashion is to make life a happiness as well as an honesty. To prescribe by letter in the dark, and to work at such a level as such practice is consonant with, is to degrade medicine and to ruin the capacity for scientific work.

